# Function and Gene Expression of Islets Experimentally Transplanted to Muscle and Omentum

**DOI:** 10.1177/0963689720960184

**Published:** 2020-12-01

**Authors:** Daniel Espes, Hanna Liljebäck, Petra Franzén, My Quach, Joey Lau, Per-Ola Carlsson

**Affiliations:** 1 Department of Medical Cell Biology, Uppsala University, Uppsala, Sweden; 2 Department of Medical Sciences, Uppsala University, Uppsala, Sweden

**Keywords:** islet transplantation, muscle, omentum, engraftment, gene expression, laser capture microdissection

## Abstract

Islet transplantation to the liver is a potential curative treatment for patients with type 1 diabetes. Muscle and the greater omentum are two alternative implantation sites, which can provide excellent engraftment and hold potential as future sites for stem-cell-derived beta-cell replacement. We evaluated the functional outcome after islet transplantation to muscle and omentum and found that alloxan-diabetic animals were cured with a low number of islets (200) at both sites. The cured animals had a normal area under the curve blood glucose response to intravenous glucose, albeit animals with intramuscular islet grafts had increased 120-min blood glucose levels. They also demonstrated an exaggerated counter regulatory response to hypoglycemia. The expression of genes important for beta-cell function was, at both implantation sites, comparable to that in native pancreatic islets. The gene expression of insulin (INS1 and INS2) and glucose transporter-2 was even increased, and the expression of lactate dehydrogenase decreased, at both sites when compared to native islets. We conclude that muscle and omentum provide excellent conditions for engraftment of transplanted islets. When compared to control, 200 islets implanted to the omentum displayed a restored glucose tolerance, whereas animals with intramuscular islet grafts of similar size displayed mild glucose intolerance.

## Introduction

Islet transplantation is a potential curative treatment for patients with type 1 diabetes and a life-saving treatment for those with severe hypoglycemic events. After the introduction of the Edmonton protocol with a steroid-free immunosuppressive regime and an increased number of transplanted islets, the outcome of islet transplantation dramatically improved^1,2^. However, there is need for improvement since still only around 50% of the transplanted patients remain insulin-independent after 5 years despite substantial immunosuppressive treatment and in most cases repeated infusions of islets from multiple donors^3^. Many of the challenges in islet transplantation are linked to the usage of the liver as implantation site. In the acute setting there is the instant blood mediated inflammatory response due to the direct contact between islets and blood^4^. In fact, in a clinical case report with islets prelabeled with the positron emission tomography tracer ^18^ F-fluorodeoxyglucose, the islets could be detected within the liver but close to 50% of the signal was immediately lost indicating massive cell destruction in the acute phase^5^. In experimental studies it has been shown that islets transplanted to the liver have a poor oxygen tension^6^ and insufficient revascularization^7^. Furthermore, the islets may suffer toxic effects from the high concentrations of immunosuppressive drugs in the portal blood due to the high uptake and metabolism of drugs in the liver^8^. It has also been shown that there is extensive formation of amyloid in islets transplanted intraportally into the liver^9,10^. Based on the challenges in intraportal islet transplantation and the need of monitoring or even retrieval possibilities of future stem-cell-derived beta-cell replacement^11^, there is an increasing interest in alternative islet implantation sites. Muscle and bone marrow have even been tested for implantation of islets in the clinical setting^12–14^ and there is currently an ongoing phase 1/2a clinical trial with allogeneic islet transplantation into the omentum (P.I. Rodolfo Alejandro, clinicaltrials.gov: NCT02213003). We have previously shown that the hypoxic phase in islets transplanted to muscle is rapidly reversed and that the islet revascularization is excellent^12,15^. Also, in experimental studies the glucose control in diabetic mice receiving intramuscular islet transplants is superior to that of animals with intraportally transplanted islets^12^. Islets transplanted to the omentum have previously been shown to engraft and cure animals in both small and large animal models^16–22^, but there have also been reports on a delayed time to normoglycemia when compared to intraportal transplantation^21^. Findings in our laboratory show that islets experimentally transplanted to the omentum have a similar time to normoglycemia as intraportally transplanted islets and a superior function for glucose control 1 month post-transplantation^23^.

In the current study we compared the function of islets transplanted to muscle and the greater omentum. We also investigated potential site-dependent changes in the gene expression of the islet grafts, which could be of detrimental importance for future trials using insulin-producing cells derived from stem cells.

## Materials and Methods

### Animals

This study was approved by the animal ethical board of Uppsala County, and all experimental procedures were conducted in accordance with the Institutional Animal Care Guidelines. Adult male C57BL/6 mice weighing ∼25 g were purchased from Taconic (Ry, Denmark). All animals were housed under standardized conditions with ad libitum access to food and water.

### Mouse Islet Isolation and Culture

Pancreatic islets from nondiabetic C57BL/6 mice were isolated using collagenase digestion and density gradient purification as previously described^24^. Islets were handpicked and cultured free-floating overnight in 5 ml RPMI 1640 medium (Sigma-Aldrich, St Louis, MO, USA) supplemented with l-glutamine (2 mmol/l; Sigma-Aldrich, St Louis, MO, USA), benzylpenicillin (100 U/ml; Roche Diagnostics, Mannheim, Germany), and 10% (vol/vol) fetal bovine serum (Sigma-Aldrich, St Louis, MO, USA). On the day of transplantation, the islets were again handpicked and manually counted. Islets with central necrosis were discarded.

### Islet Transplantation

Two hundred mouse islets were transplanted under aseptic conditions into the abdominal muscle or omentum of recipient nondiabetic or alloxan-diabetic C57BL/6 mice. The animals were anesthetized with an intraperitoneal injection of Avertin (0.02 ml/g), a 2.5% solution of 10 g 97% 2.2.2-tribromoethanol (Sigma-Aldrich, St Louis, MO, USA) in 10 ml 2-methyl-2-buthanol (Kemila, Stockholm, Sweden). Islets were transplanted to the abdominal muscle as previously described^15^. In order to transplant islets to the greater omentum, a midline incision in the skin and abdominal muscle was made and the stomach was exposed. The greater omentum was localized and carefully extended by holding the omental fat and a ligature was placed, but not fixed, around the omentum. An opening between the sheets of the omentum was made with a cannula, a braking pipette filled with the islets was inserted, the islets were infused into the pouch, and the ligature was thereafter closed. The abdominal muscle was carefully sutured followed by suturing of the skin.

### Induction of Diabetes and Functional Tests After Islet Transplantation

Diabetes was induced in C57BL/6 mice by a single intravenous injection of alloxan (75 mg/kg, Sigma-Aldrich, St Louis, MO, USA) 3–5 days prior to transplantation. Diabetes in mice was defined as repeated blood glucose concentrations exceeding 15 mmol/l, as measured by blood glucose reagent strips (Freestyle Lite, Abbot, Alameda, CA, USA) on two consecutive days. Blood glucose concentrations were monitored daily for the first week post-transplantation and then every fifth day. Thirty days post-transplantation an intravenous glucose tolerance test (IVGTT, 2 g/kg) was performed in cured non-fasted animals (cured was defined as a non-fasting blood glucose concentration <12 mmol/l for two consecutive measurements on separate days). Blood glucose concentrations were monitored for 2 h following glucose injection. On the following day an intravenous insulin tolerance test (ITT; 2 U/kg NovoRapid, Novo Nordisk, Bagsvaerd, Denmark) was performed during which blood glucose concentrations were monitored for 2 h. Nondiabetic C57BL/6 mice were used as controls for both the IVGTT and the ITT. When the animals thereafter were killed, the pancreas was surgically removed, placed in acid ethanol [0.18 mol/l HCl in 95% (vol/vol) ethanol], sonicated in order to disrupt the cells and thereafter frozen. The remaining insulin content in the pancreas was measured with an insulin enzyme-linked immunosorbent assay (Mercodia, Uppsala, Sweden) in order to exclude endogenous beta-cell regeneration. An insulin content >10% of that of the pancreas of control nondiabetic animals was used as an exclusion criterion for the study.

### Preparation of Tissue for Laser Microdissection

For the gene expression studies nondiabetic recipient animals were used. The animals were anesthetized with an intraperitoneal injection of pentobarbital (60 mg/kg; Apoteket, Gothenburg, Sweden) 30 days post-transplantation, and the muscle or omentum containing transplanted islets as well as the pancreas were surgically excised under sterile conditions and quickly embedded in frozen section medium (Richard-Allan Scientific NEG 50, Thermo Scientific, Kalamazoo, MI, USA) and snap frozen in liquid nitrogen. Frame slides (POL-Membrane 0.9 µm, Leica Microsystems, Wetzlar, Germany) were exposed to UV light overnight for cross-linking of the membrane in order to improve excision of islet tissue on the membrane. Sections were simultaneously stained with hematoxylin to confirm the presence of islets and when confirmed, then consecutive sections (10 µm) were mounted on frame slides, fixed in ice cold acetone for 2 min, and thereafter dried with cold air before storage in RNase free 50 ml tubes (Ambion, Life Technologies Europe BV, Stockholm, Sweden) in −80°C. The frame slide with sections was thawed for 30 s before hydration in nuclease free water (Ambion, Stockholm, Sweden). Staining was performed using RNase free hematoxylin (Arcturus HistoGene Staining Solution, Applied Biosystems, Foster City, CA, USA). Laser microdissection of the islets and islet grafts was performed with a Leica LMD6000 microscope using the Leica Microdissection System software version 7.5.1.5250 (Leica Microsystems, Wetzlar, Germany). Approximately (1.5–3.0) × 10^6^ µm^2^ of islet tissue was collected for each sample.

### RNA Isolation

Total RNA was isolated from the laser microdissected samples according to the manufacturer’s instructions (RNeasy Plus Micro Kit, Qiagen, Hilden, Germany). This kit contains a gDNA Eliminator spin column, which will remove the genomic DNA. The amount and purity [optical density (OD) 260/280] of the total RNA was determined using a NanoDrop 2000C spectrophotometer (Thermo Scientific, Waltham, MA, USA). Yields of the LMD samples were in the range of 5–15 ng RNA. All RNA samples had OD 260/280 between 1.9 and 2.1, which is in the range for pure RNA. The extracted total RNA was dissolved in nuclease free water and stored at −80°C until cDNA synthesis. The RNA was transcribed to cDNA by Superscript First-Strand Synthesis Super Mix for quantitative-real time polymerase chain reaction (qRT-PCR; Invitrogen, Life Technologies, Stockholm, Sweden) according to the manufacturer’s instructions. Two independent reverse transcriptase reactions were carried out for each RNA sample. The cDNA was amplified using RealTime ready cDNA Pre-Amp Master kit (Roche Diagnostics, Mannheim, Germany) for the genes of interest (Table 1).

For primer sequences used in this study, see Supplemental Table 1. All primers, except for *INS1*, *INS2*, and *HPRT*, were purchased from Sigma-Aldrich (St Louis, MO, USA). Primers for *INS1*, *INS2*, and *HPRT* were purchased from Tebu-Bio (Roskilde, Denmark); Catalog no. *INS1*; MQP027447, *INS2*; MQP027448, and *HPRT*; MQP030898.

### Quantitative Real-time PCR

The qPCR assay was performed using a Light Cycler 480t (Roche Diagnostics, Mannheim, Germany) and Light Cycler FastStart DNA Master PLUS SYBR Green I kit (Roche Diagnostics, Mannheim, Germany) for detection. All qPCR samples were run in duplicates. Moreover, cDNA was prepared twice from each RNA sample and thereafter each cDNA was amplified twice with the primer pool to ensure inter-run specificity.

To determine the PCR efficiency, the primer pairs were analyzed using a dilution curve with 10-fold cDNA template dilutions between 5 and 0.05 ng/µl. The efficiency was calculated using the formula: Efficiency = −1 + 10^(−^^1/slope)^. The expression stability of reference transcripts *GAPDH*, *RPS7*, and *HPRT* was evaluated using the Normfinder software^25^. The analysis indicated that the best normalization was obtained by using the geometric mean of the expression of the reference genes *GAPDH* and *HPRT*. This normalization was therefore used to compare the islet-specific transcripts in all samples. The cycle threshold (Ct) values were used to calculate the amount of PCR product compared to reference genes by subtracting the Ct value for reference genes from the Ct value for the gene studied (ΔCt). Relative mRNA expression was calculated as 2^−ΔCt^ and data are presented as relative gene expression. For each transplantation site, that is, muscle and omentum, five animals were used and the LMD islet graft material was pooled for the respective site in order to get enough material for three LMD samples. Control samples derived from native pancreatic islets were dissected from two separate animals.

### Agarose Gel Electrophoresis

To confirm amplicon size, qPCR products were analyzed by electrophoresis using a 3% agarose gel (PCR-grade, Bio-Rad, Hercules, CA, USA). The PCR products were mixed with five times loading buffer (Bio-Rad, Hercules, CA, USA) before loading. A 50 bp ladder (Invitrogen, Carlsbad, CA, USA) was used to determine the size of the PCR products. Electrophoresis was conducted using an electrical field of 5 V/cm for 80 min and the bands were visualized using GelRed (Biotium, Hayward, CA, USA) and detected using the Chemi Doc MP Imaging System (Bio-Rad, Hercules, CA, USA).

### Immunohistochemistry

Insulin was stained on the cryosections using an anti-insulin primary antibody (guinea pig polyclonal, dilution 1:400; incubated at 4°C overnight, Fitzgerald, Acton, MA, USA), which was detected by Alexa Fluor 488 (goat anti-guinea pig, dilution 1:1000, Invitrogen, Carlsbad, CA, USA) secondary antibody. ProLong Gold Antifade reagent with 4′,6-diamidino-2-phenylindole (Life Technologies, Rockville, MD, USA) was used for mounting and nuclei staining. From separate animals, islet grafts from muscle and omentum and pancreas were fixed in 4% (vol/vol) paraformaldehyde and embedded in paraffin and sectioned at a thickness of 4 µm. The islet architecture was characterized on paraffin sections, which were double stained for insulin (dilution 1:400; Fitzgerald, Acton, MA, USA) and glucagon (mouse monoclonal, dilution 1:800, Abcam, Cambridge, UK). Secondary antibodies used were Alexa Fluor 488 (goat anti-guinea pig) and Alexa Fluor 594 (donkey anti-mouse; dilution 1:250; Invitrogen, Carlsbad, CA, USA). Nuclei were stained with Hoechst.

Light microscopy images were acquired with a Leica LMD6000 laser microdissection microscope (Leica Microsystems, Wetzlar, Germany). Fluorescent immunohistochemistry images were acquired with Zeiss LSM780 (Zeiss, Jena, Germany) confocal. The percentage of alpha- and beta-cell area was calculated as the percentage of the respective glucagon and insulin positive area divided by the combined area of insulin and glucagon for that islet/islet graft. The respective areas of insulin and glucagon were calculated by using the image software Imaris (Bitplane AG, Zurich, Switzerland) with a fixed intensity cut-off value for each staining. The percentage of each image was calculated as an average for each animal and considered as one experiment.

### Glycogen Assay

Glycogen concentrations were measured in 10 mg homogenized muscle and liver tissue from cured transplant recipients (*n* = 3 for each site) and control animals (*n* = 4). Glycogen Assay Kit II (Abcam, Cambridge, UK) was used according to the manufacturer’s instructions. Reading of the plate was performed using Spark Microplate Reader (Tecan, Männedorf, Switzerland).

### Statistical Analysis

GraphPad Prism version 6.07 (GraphPad Software, La Jolla, CA, USA) was used for statistical analysis. For comparison of relative gene expression data between the three groups, a nonparametric one-way analysis of variance (ANOVA) was applied with Dunn’s post hoc test using native pancreatic islets as control. For comparison of blood glucose measurements between the three groups, a one-way ANOVA with Tukey’s post hoc test was applied. Comparison of time to normoglycemia was performed as a survival curve using Log-rank test (Mantel-Cox). For all statistical analysis, *P* values less than 0.05 were considered significant and all values are given as means ± standard error of the mean.

## Results

### Islet Graft Function

**Figure 1.**
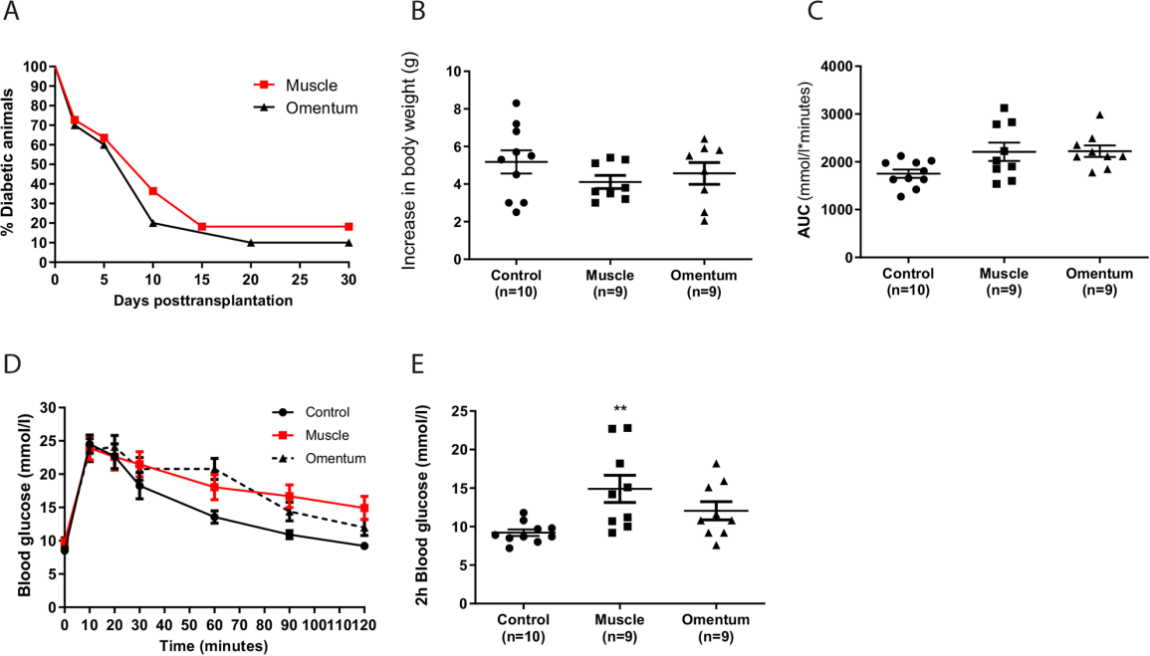
Time to normoglycemia and islet function after transplantation to muscle or the omentum. Two hundred islets were transplanted to either the abdominal muscle or the greater omentum in alloxan-diabetic C57BL/6 mice. (A) The time to normoglycemia was similar for both implantation sites (median 10 days). Nine out of 11 animals were cured by islets transplanted into muscle and 9/10 animals with islets transplanted to the omentum. (B) The body weight development was similar in all cured transplanted animals when compared to nontransplanted nondiabetic control animals. (C, D) One month post-transplantation all cured animals were subjected to an IVGTT. The overall response to the IVGTT, expressed as an AUC, was similar in the transplanted animals for both implantation sites when compared to control animals. (E) The blood glucose levels 2 h after the intravenous glucose injection was increased in the animals with intramuscular islet grafts when compared to control animals, but not when compared to animals with islets transplanted to the omentum. **P < 0.01 when compared to control animals. Data are expressed as means ± SEM for 9–10 animals. AUC: area under the curve; IVGTT: intravenous glucose tolerance test; SEM: standard error of the mean.

Syngeneic transplantation of 200 islets reversed diabetes in 9 out of 11 (82%) animals after intramuscular implantation and in 9 out of 10 (90%) after implantation into the omentum. The time to normoglycemia did not differ between the implantation sites; median time to reversal of diabetes (<12 mmol/l) was 10 days for both groups (Fig. 1A). The feeding behavior and physical activity level were observed to be normal in all transplanted animals. The body weight development in the cured animals was comparable to that of nontransplanted control animals (Fig. 1B). Thirty days post-transplantation, all cured transplanted animals, as well as 10 nondiabetic control mice, were subjected to an IVGTT. No difference in glucose control during the IVGTT was observed between the three groups when the data were expressed as an area under the curve (Fig. 1C). However, the blood glucose levels 2 h after injection of glucose were higher in the animals with intramuscular islet grafts as compared to control mice (Fig. 1E). In fact, six out of nine animals with intramuscular islet transplants and four out of nine animals with islets transplanted to the omentum had a 2-h blood glucose level >11.1 mmol/l.

**Figure 2.**
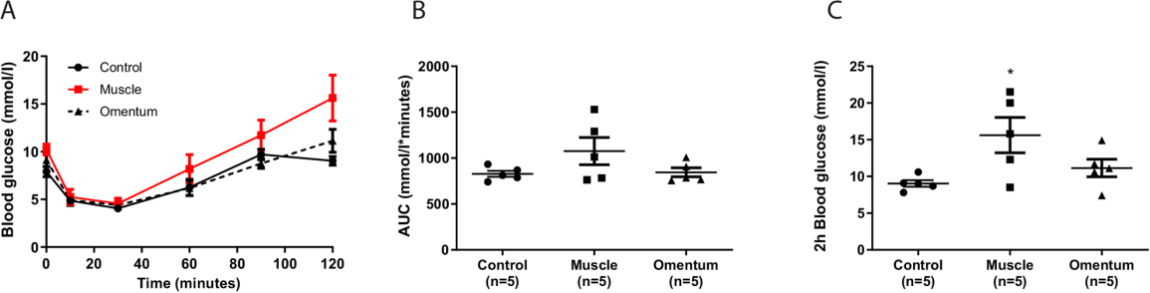
ITT in alloxan-diabetic animals cured by islet transplantation to muscle or omentum. One month post-transplantation cured transplanted animals were subjected to an ITT and compared to nondiabetic nontransplanted control animals. (A) The initial response to a bolus of intravenous insulin (2 U/kg) was similar in all transplanted animals when compared to control. (B) The overall response to the ITT, expressed as an area under the curve, was similar in the transplanted animals when compared to control animals. (C) The blood glucose levels 2 h after the insulin bolus were increased in the animals with intramuscular islet grafts. *P < 0.05. Data are expressed as means ± SEM for five animals in each group. ITT: insulin tolerance test; SEM: standard error of the mean.

In a subgroup of the cured transplanted animals (*n* = 5 for each implantation site) an ITT was performed, which showed similar nadir values of blood glucose in both groups of transplanted animals and in control animals (Fig. 2A, B). However, as for the IVGTT, the blood glucose level 2 h postinjection was increased in the animals with intramuscular islet transplants (Fig. 2C).

### Islet Composition After Transplantation

**Figure 3.**
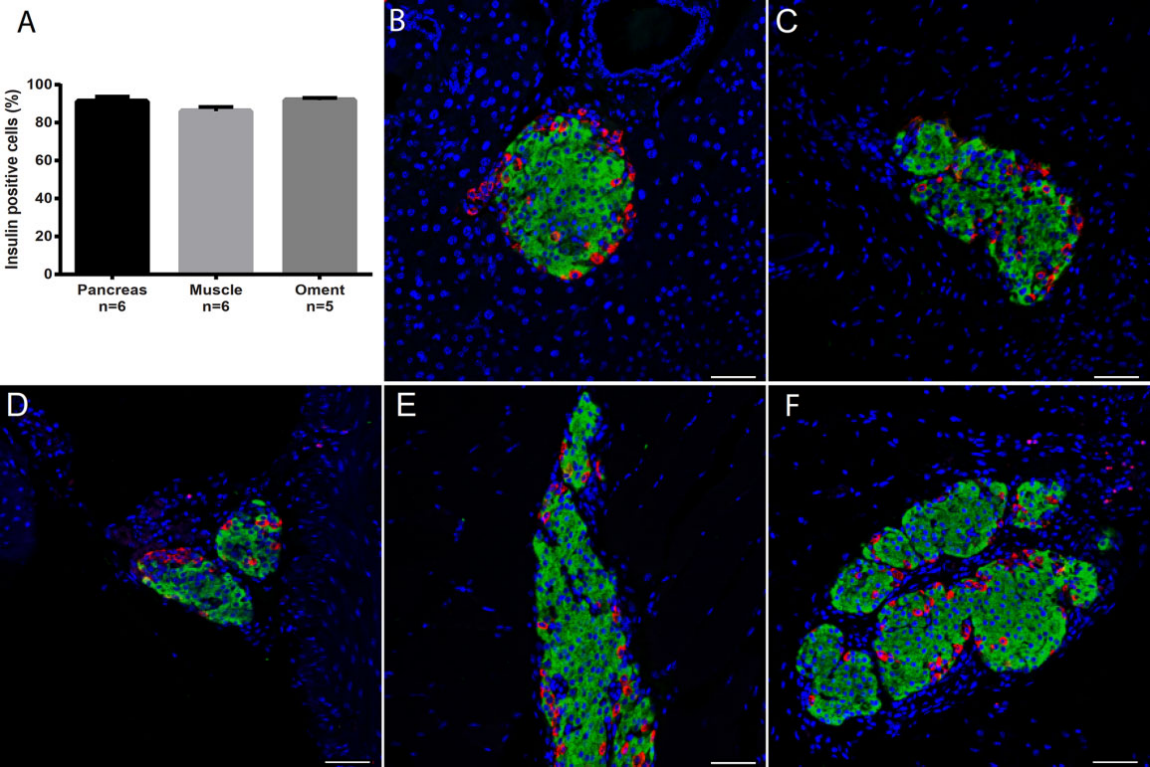
Cellular composition of islets implanted in muscle or in omentum when compared to native islets. Paraffin embedded sections were stained for insulin (green) and glucagon (red) and the total area of cells positive for the respective hormone was assessed. (A) The percentage of insulin positive cells, that is, beta cells, was calculated by dividing the insulin area with the total area of insulin and glucagon. No difference was observed between islets at the two implantation sites and native islets. Data are expressed as means ± SEM for five to six animals. (B) Immunohistochemistry of native pancreatic islet: alpha cells were mainly found in the islet periphery. Immunohistochemistry of transplanted islets found as single islet in muscle (C) or omentum (D) show very similar architecture to that of native islets. Even when the transplanted islets occurred as clusters the alpha cells were mainly found in the individual islet periphery in both muscle (E) and omentum (F). Nuclei are stained with Hoechst (blue). Scale bar indicates 50 µm. SEM: standard error of the mean.

The composition of the transplanted islets was observed to be normal when compared to native pancreatic islets. The percentage of insulin (Fig. 3A) and glucagon (not shown) positive cells was unaltered in both islets transplanted to muscle and the omentum. In separate transplanted islets, both in muscle and the omentum, the alpha cells were found preferentially in the periphery, as in native pancreatic islets (Fig. 3B–D). Likewise, in islets that had formed clusters after transplantation, the composition of alpha and beta cells was maintained as in individual islets (Fig. 3E, F).

### Gene Expression Analysis

**Figure 4.**
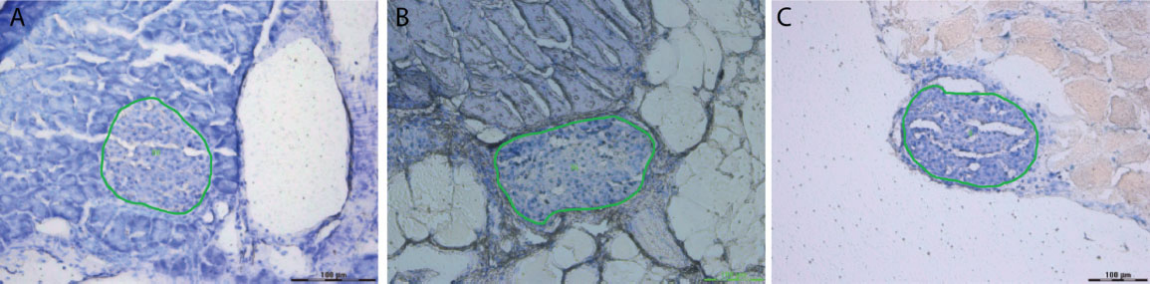
Hematoxylin staining of snap frozen tissue prepared for laser capture microdissection. Pancreata from nondiabetic animals and islet grafts in muscle or omentum were snap frozen in liquid nitrogen. Cryosections were prepared at a thickness of 10 µm on frame slides, which were then quickly stained with RNAse free hematoxylin. Islets could easily be detected in both the native pancreas (A), in the muscle (B) and in the omentum (C). The encircled area in each image was dissected by laser and captured in lysis buffer for further isolation and amplification of mRNA. Scale bar indicates 100 µm.

**Figure 5.**
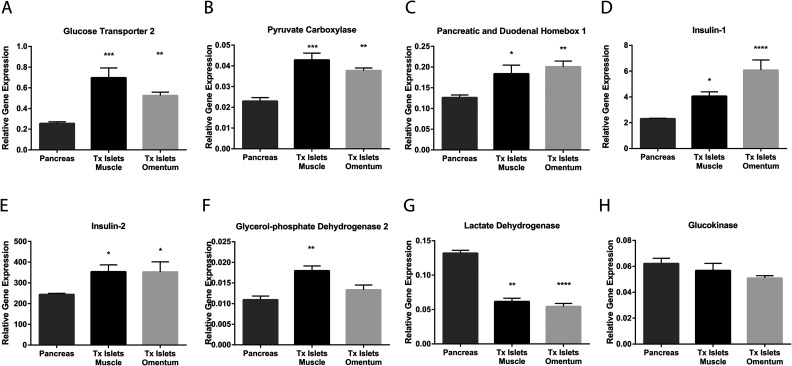
Relative gene expression levels in native pancreatic islets and in islet grafts retrieved by laser capture microdissection from muscle or the omentum of nondiabetic recipients. Relative gene expression for *GLUT2* (A), *PCX* (B), *PDX1* (C), *INS1* (D), *INS2* (E), *GPD2* (F), *LDHA* (G), and *GCK* (H) in native pancreatic islets (dark gray bars), in intramuscular islet grafts (black bars), and in islets transplanted to omentum (light gray bars) 1 month post-transplantation. Values are normalized to the geometric mean of the reference genes *GAPDH* and *HPRT.* *P < 0.05, **P < 0.01, ***P < 0.001, and ****P < 0.0001 compared to native pancreatic islets. Data are expressed as means ± SEM for 8–12 experiments. SEM: standard error of the mean.

The native pancreatic islets and islet grafts could easily be detected with hematoxylin staining using the Leica LMD6000 laser microdissection microscope (Fig. 4A–C). An upregulated expression of *GLUT2*, *PCX*, *PDX1*, *INS1*, and *INS2* was observed for both islets transplanted to muscle and omentum when compared to native pancreatic islets (Fig. 5A–E). In islets transplanted to muscle an upregulation of *GPD2* was also observed (Fig. 5F). In contrast, the expression of *LDHA* was found to be downregulated in both islets transplanted to muscle and omentum compared to native islets (Fig. 5G), whereas the expression of *GCK* was unaltered (Fig. 5H).

### Glycogen Concentration

**Figure 6.**
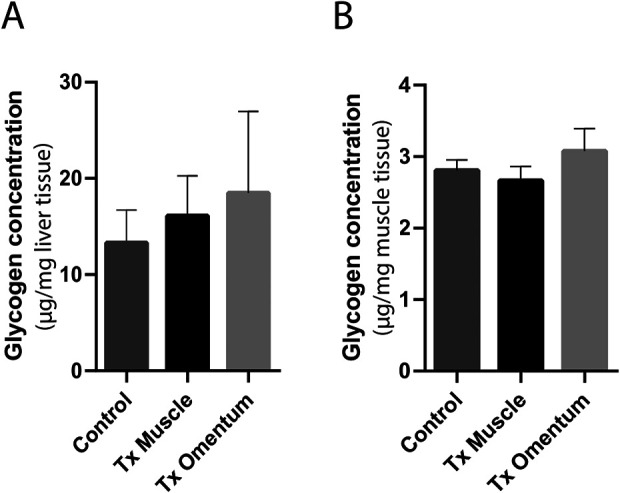
Glycogen concentrations in liver and striated muscle were assessed in cured transplanted C57BL/6 mice 30 days post-transplantation. The glycogen levels in liver and muscle were similar in the transplanted animals (*n* = 3 for both sites) and controls (*n* = 4) (A, B). The glycogen concentration is given in µg glycogen per mg homogenized tissue of liver or muscle, respectively. Data are expressed as means ± SEM. SEM: standard error of the mean.

Glycogen content was measured in striated muscle (contralateral to graft) and liver to audit if there were any differences in glycogen synthesis, depending on portal or systemic insulin release from islet graft transplanted to either omentum or muscle, of the animals. There was no difference in glycogen content in either liver or striated muscle between animals transplanted with islets to the omentum or muscle (Fig. 6). Similarly, the glycogen content of the organs did not differ between control animals (nondiabetic, nontransplanted) and the islet transplanted animals (Fig. 6).

## Discussion

The present study shows that insulin-deficient diabetes in mice can be efficiently reversed by transplantation of only 200 islets to striated muscle or the greater omentum. For both implantation sites at least 80% of the animals were cured after receiving 200 islets. However, animals with intramuscular islet grafts displayed elevated blood glucose levels 120 min after a glucose challenge, which could reflect either a marginal surviving islet mass, dysfunctional islets, or the systemic instead of portal drainage of these islets.

Albeit islets implanted to the intramuscular site as into the omentum are rapidly revascularized with a restoration of the vascular network^12,23^, we and others have previously reported of substantial fibrosis formation in intramuscular islet grafts indicative of early cell death^26,27^. Indeed, our recent studies also show that intramuscularly transplanted islets suffer from prevailing hypoxia in the first week after transplantation with concomitantly increased cellular death^15^. However, site-dependent gene expression changes causing islet graft dysfunction are also known to occur in the liver (have been shown in both experimental and clinical studies)^28–30^, the pancreas^28^, and beneath the renal capsule^31^, although this has not been characterized for the presently used implantation sited. The expression of several genes important for maintaining the beta-cell phenotype, such as *PDX1*, and for function, such as *GCK*, was decreased in both intraportally and intrapancreatically transplanted islets^28^, and *INS1* as well as *INS2* was decreased in renal subcapsular grafts^31^. Interestingly, no such detrimental changes were observed for either islets in the muscle or greater omentum in the present study. This could potentially be a very important feature in favor of these two implantation sites when moving forward with clinical trials using stem-cell-derived insulin-producing cells. For both implantation sites there was an upregulated expression of *GLUT2*, *PCX*, *PDX1*, *INS1* as well as *INS2* when compared to native islets. This could reflect an upregulation due to the increased demands of a marginal islet mass. However, we cannot rule out that the samples obtained from endogenous pancreas could contain small amounts of contaminating exocrine tissue. Moreover, in contrast to previous reports of islets grafted to the pancreas or beneath the renal capsule^28,31^, the gene expression of *LDHA* was even decreased at both the intramuscular and omental sites when compared to native islets. Particularly for intramuscular islet grafts this latter findings may be important, since an increased expression of *LDHA* would likely have cause the transplanted beta cells to secrete insulin not only in response to ambient glucose levels but to generated lactate^32^. Indeed, exercise have previously been shown to induce hypoglycemia in animals with islets transplanted beneath the renal capsule^33^. All in all, there were no signs of beta-cell dysfunction in the grafts and it seems most likely that the glucose intolerance reflects a marginal islet mass at the intramuscular site.

Although both muscle and the greater omentum provide excellent conditions for revascularization, their venous drainage is different. Muscle has a systemic drainage, whereas the omentum as native islets has a portal drainage. Since the hepatic cells are a major target for insulin, the importance of portal drainage has been one of the main arguments for using the liver as an islet implantation site. It has also been shown that a systemic venous drainage of islet grafts increases the systemic levels of circulating insulin despite similar glucose levels, which is suggestive of an insulin-resistant state^34^. The C-peptide levels were, however, not increased in the animals with islet grafts with systemic drainage, which indicates that the secretion of insulin was not altered but rather the clearance of insulin^34^. Since insulin or C-peptide levels were not measured in the current study, it cannot be excluded that the reduced clearance of glucose during the IVGTT in the animals with intramuscular islet grafts in part reflected an insulin-resistant state. However, during the ITT the initial glucose lowering response was identical to control in both groups of transplanted animals, which at least is suggestive of a normal response to insulin in peripheral tissue. This is also supported by the finding of similar glycogen levels in liver and muscle irrespective of transplant site.

Noteworthy, the blood glucose levels 2 h after the insulin injection was increased in the animals with intramuscular islet grafts when compared to control, suggestive of an exaggerated counter regulatory response to hypoglycemia. The reason for this is presently obscure, since, for example, no glucagon concentrations were measured^31^. However, previous studies indicate that the glucagon response to hypoglycemia may be site dependent. Animals with intraportal islet transplants subjected to hypoglycemia had a blunted glucagon response, whereas animals with islets transplanted beneath the kidney capsule or to the intraperitoneal cavity had a normal response^35^. Clinically, the finding at the intraportal site has since then been debated, since early studies showed that the glucagon response to insulin-induced hypoglycemia is blunted and that a rise in glucagon secretion only occurs after arginine stimulation^36^, whereas a more recent study reported that the glucagon secretion does increase in response to insulin-induced hypoglycemia, although not to the same extent as in healthy controls^37^. Nevertheless, in contrast to our previous observations of decreased percentages of glucagon-positive cells for islets experimentally transplanted to the liver^38^, we found that the islet composition is unaltered in both islets transplanted to muscle and to the omentum.

Both muscle and the greater omentum has in experimental studies from our group been shown to be superior to the liver as islet implantation sites^12,23^. However, these two sites have never been compared head-to-head regarding islet graft function. In the current study we report that alloxan-diabetic mice can be cured with a low number of islets (200) by transplantation to either muscle or the greater omentum. The restoration of normoglycemia is rapid and the animals respond well to an IVGTT and can effectively counteract insulin-induced hypoglycemia regardless of implantation site. Moreover, in contrast to previous experimental studies of islets grafted to the liver, beneath the renal capsule or into the pancreas, we observe no detrimental expression changes in genes vital for beta-cell function. These findings support the use of both muscle and the omentum as implantation site for islet transplantation, although differences in the human omentum composition and the need of a much larger islet mass may impact results in the clinical setting. The fact that the expression of genes important for maintaining a beta-cell phenotype is sustained or even augmented at both sites could be of great importance when considering implantation sites for future clinical transplantation of stem-cell-derived insulin-producing cells.

## Supplemental Material

Supplemental Material, sj-pdf-1-cll-10.1177_0963689720960184 - Function and Gene Expression of Islets Experimentally Transplanted to Muscle and Omentum Click here for additional data file. Supplemental Material, sj-pdf-1-cll-10.1177_0963689720960184 for Function and Gene Expression of Islets Experimentally Transplanted to Muscle and Omentum by Daniel Espes, Hanna Liljebäck, Petra Franzén, My Quach, Joey Lau and Per-Ola Carlsson in Cell Transplantation
