# Supplementary material for: Function and Gene Expression of Islets Experimentally Transplanted to Muscle and Omentum
Source: Cell Transplant. 2020 Dec 1;29:0963689720960184. doi: 10.1177/0963689720960184 (PMC8544762; doi:10.1177/0963689720960184)
Supplement: Supplemental Material, sj-pdf-1-cll-10.1177_0963689720960184 - Function and Gene Expression of Islets Experimentally Transplanted to Muscle and Omentum [file sj-pdf-1-cll-10.1177_0963689720960184.pdf]

Supplementary Table 1

Primer data.

**Gene**	**Oligo**	**Sequence (5´-3´)**	**Amplicon size (bp)**
GCK	forward<br>reverse	CCCTGTAAGGCACGAAGACA<br>AGTCCCACGATGTTGTTCCC	96
GLUT2	forward<br>reverse	TCGCCTCATTCTTTGGTGGG<br>ATCCCATCAAGAGGGCTCCA	102
GPD2	forward<br>reverse	GATCCTGACTCCCTTGCTCG<br>ATATGCCAGGCTCACTTGCT	176
INS1	forward<br>reverse	MQP027447<br>Tebu-Bio, Roskilde, Denmark	141
INS2	forward<br>reverse	MQP027448<br>Tebu-Bio, Roskilde, Denmark	105
LDHA	forward<br>reverse	GCAGACAAGGAGCAGTGGAA<br>ATGGCCCAGGATGTGTAACC	92
PCX	forward<br>reverse	GCATTGAGGTTTTCCGGAGTG<br>GTGCAATGACCTTGACGAGC	117
PDX1	forward<br>reverse	CCTTTCCCGAATGGAACCGA<br>TTCCGCTGTGTAAGCACCTC	132
GAPDH	forward<br>reverse	TGGTGAAGCAGGCATCTGAG<br>TGAAGTCGCAGGAGACAACC	78
HPRT	forward<br>reverse	MQP030898<br>Tebu-Bio, Roskilde, Denmark	152
RPS7	forward<br>reverse	TGAAGCCATGTTCAGCTCG<br>CTTTCCGACCACCACCAACTT	182
